# Functional constituents of plant-based foods boost immunity against acute and chronic disorders

**DOI:** 10.1515/biol-2022-0104

**Published:** 2022-09-08

**Authors:** Waseem Khalid, Muhammad Sajid Arshad, Muhammad Modassar Ali Nawaz Ranjha, Maria Barbara Różańska, Shafeeqa Irfan, Bakhtawar Shafique, Muhammad Abdul Rahim, Muhammad Zubair Khalid, Gholamreza Abdi, Przemysław Łukasz Kowalczewski

**Affiliations:** Department of Food Science, Government College University, Faisalabad, 38000, Pakistan; Institute of Food Science and Nutrition, University of Sargodha, Sargodha, 40100, Pakistan; Department of Food Technology of Plant Origin, Poznań University of Life Sciences, 60-624 Poznań, Poland; Department of Biotechnology, Persian Gulf Research Institute, Persian Gulf University, Bushehr, 75169, Iran

**Keywords:** acute diseases, antioxidants, chronic diseases, immunity booster, nutraceuticals, phenolic compounds, minerals, vitamins

## Abstract

Plant-based foods are becoming an increasingly frequent topic of discussion, both scientific and social, due to the dissemination of information and exchange of experiences in the media. Plant-based diets are considered beneficial for human health due to the supply of many valuable nutrients, including health-promoting compounds. Replacing meat-based foods with plant-based products will provide many valuable compounds, including antioxidants, phenolic compounds, fibers, vitamins, minerals, and some ω3 fatty acids. Due to their high nutritional and functional composition, plant-based foods are beneficial in acute and chronic diseases. This article attempts to review the literature to present the most important data on nutrients of plant-based foods that can then help in the prevention of many diseases, such as different infections, such as coronavirus disease, pneumonia, common cold and flu, asthma, and bacterial diseases, such as bronchitis. A properly structured plant-based diet not only provides the necessary nutrients but also can help in the prevention of many diseases.

## 1 Plant-based diets and foods

Plant-based diets are gaining more and more popularity, especially among adolescents and women. Reasons for changing eating habits are different perceptions of the earth’s resources and the environment, ethical issues related to animal care, the use of antibiotics and growth promoters in animal production, and the threat of zoonoses (especially in the light of the persistent SARS-CoV-2 epidemic), and possible health benefits resulting from the multitude of nutritional and health-promoting compounds present in plant-based foods [1,2,3,4]. Published research has shown that reducing the risk of serious chronic diseases, such as obesity, heart disease, and type 2 diabetes, is possible by replacing meat products with plant-based foods [5,6]. However, it should be highlighted that a poorly constructed plant-based diet can also cause many deficiencies of important nutrients that are not absorbed by human organism due to the elimination of animal products from the diet [3].

**Figure 1:**
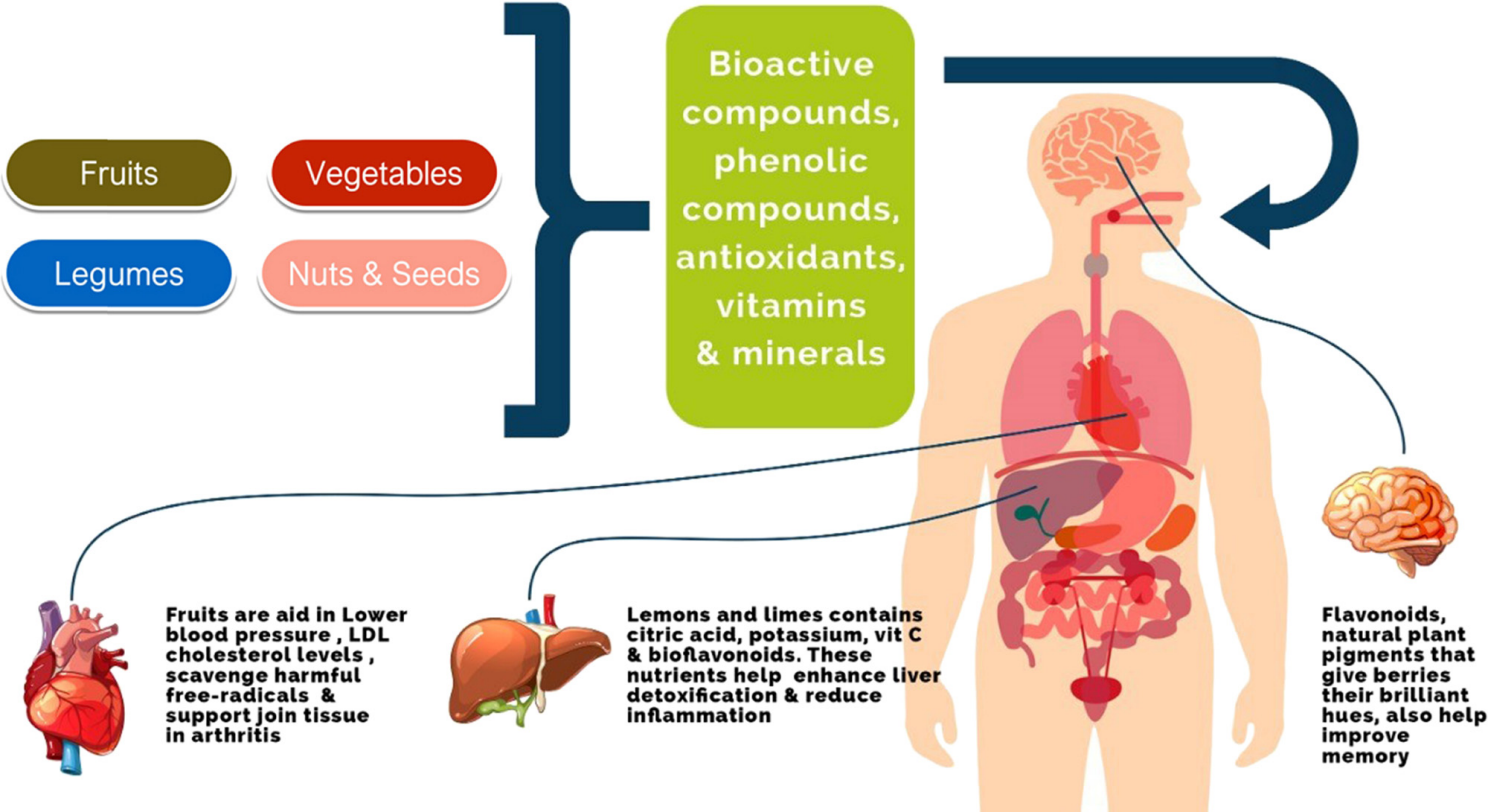
Different types of plant-based foods and their potential benefits on human organs.

Plant-based foods include fruits, vegetables, nuts, seeds, whole grains, legumes, and beans [7]. These products contain bioactive secondary metabolites comprehensively distributed in plants and are predominantly manufactured by the shikimic acid pathway [8]. Legume phytochemicals are categorized into different groups and contain compounds, such as lignans, flavonoids, stilbenes, and phenolic acids [9,10]. Plants and their by-products contain phenolic acids, such as flavanols, condensed tannins, and anthocyanins, are the primary classes of polyphenols and are mainly present in legumes. Flavonoids, phenolic acids, and procyanidins are the prevailing phenolic compounds present in peas, customary beans, and lentils [11]. The bioavailability and bio-accessibility of starch, lipids, proteins, and functional phytochemicals (carotene) are acknowledged as secondary indicators of the digestibility of plant-based food. Figure 1 shows the effects of different types of plant-based foods on the human body organ.

The functional components are produced from plant-based food (vitamins C, D, and E) or absorbed from the environment (i.e., zinc and magnesium) that aid in improving immunity against viral diseases [12]. Bioactive compounds, including polyphenols, ensure an authentic beneficial therapeutic potential as these are promoted within the direction of the inhibitor activities [13]. The ingestion of plant-based foods is considerably related to the lower risk of coronary artery diseases, cancer, diabetes, and Alzheimer’s disease (AD). The major types of plant-based food and their parts are listed in Table 1.

**Table 1:** Selected plant-based food characteristics

Plant food sources	Types	Functional ingredient	Part used	References
Vegetable	Green leafy: kale, spinach, and lettuce	Fibers, antioxidants, polyphenols, vitamins, minerals, carotenoids, and fatty acids	Leaves, stems, root, pods, sprouting, axillary buds, flower, shoots, and fruits	[167,168,169,170,171,172,173,174]
	Root: radishes, potato, beets, sweet potato, garlic, and yam			
	Cruciferous: cauliflower, broccoli, brussels sprouts, and cabbage			
	Marrow: pumpkin, zucchini, and cucumber			
	Plant stem (edible): celery and asparagus			
	Allium: garlic, onion, and shallot			
Fruit	Citrus: limes, grape fruits, mandarins, and oranges	Antioxidants, fatty acids, fibers, vitamins, minerals, polyphenols, and carotenoids	Peel, pulp, fruit, flower, and seed	[100,175,176,177,178,179,180,181,182]
	Tropical and exotic: apples, pears, mangoes, avocados, bananas, and tomatoes			
	Stone fruit: peaches, apricots, plums, and nectarines			
	Berries: blueberries, raspberries, strawberries, kiwifruit, and passion fruit			
	Melons: melons, honeydew melons, watermelons, and rockmelons			
	Dry fruits: almond, hazelnuts, walnuts, pistachios, raisins, and cashew			
Cereal	Wheat, oats, rye and barley, sorghum, rice, and maize	Phenolic acids, flavones, phytic acid, flavonoids, coumarins, and terpenes	Grain, bran, endosperm, and germ	[183,184,185,186,187]
		Ferulic acid, phytic acid, glutathione, and phytosterols		
		Vitamins, minerals, and fibers		
Beans/legume	Dried beans and peas: Red kidney beans, haricot beans, lentils, dry beans, snap beans, shell beans, and chickpeas	Bioactive compounds (phytochemicals and antioxidants, isoflavones, lignans, protease inhibitors, trypsin and chymotrypsin inhibitors, saponins, alkaloids, phytoestrogens, and phytates)	Grain	[188,189,190,191,192]
	Fresh beans and peas: Butter beans, green peas, broad beans, green beans, soybeans, and snow peas			
	Types of legumes: Peanuts, black-eyed peas, chickpeas, and Lentils			

## 2 Natural components of plant-based foods as an immune booster

**Figure 2:**
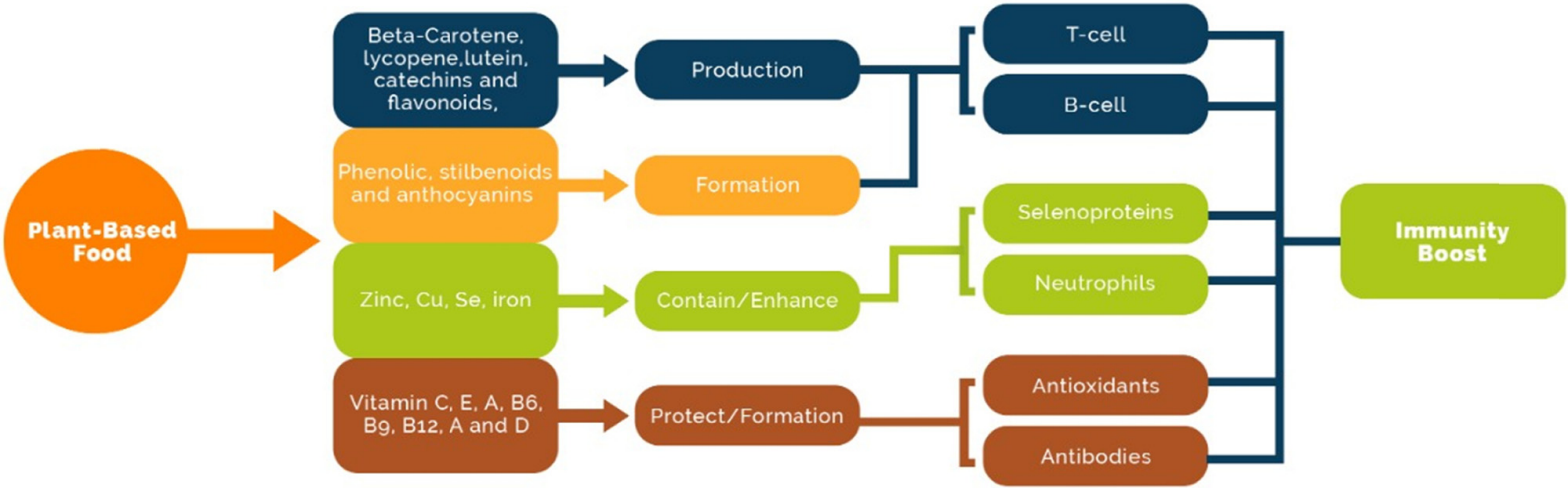
Plant-derived natural components that aid in boost immunity.

The immunity system is a complicated system of cells and proteins that protect the body from infection. The immunity system retains a history of each type of bacteria (microorganism) that has been destroyed, so if the microorganism enters the body again, it can quickly identify and destroy the microorganism [14]. The main components of the immunity system are white blood cells, antibodies, complement system, lymphatic or drainage system, organs, such as spleen, bone marrow, and gland-like thymus. The nutrients from fruits and vegetables (such as β-carotene, vitamin C, and vitamin E) can enhance immune function. Meanwhile, several vegetables, fruits, and some plant foods are also enriched in antioxidants, so they aid in alleviating oxidative strain [15]. Plant-derived natural components that aid in boosting immunity are shown in Figure 2.

### 2.1 Vitamins

#### 2.1.1 Vitamin C

Vitamin C is an important micronutrient for human beings, and its multi-effect function is associated with its capacity to provide electrons. It is an effective antioxidant and cofactor for biosynthesis and gene regulation enzymes. It is an antioxidant that assists in destroying free radicals and regulating the body’s natural immune reaction. Vitamin C sources include red capsicum, tangerine, red strawberries, kiwi, green broccoli, mango, lime, citrus fruits, tomato juice, and potatoes [16,17].

Vitamin C promotes immune attack by assisting multiple cell functions of the innate and adaptive immune systems. It maintains the function of the epithelium and stimulates the action of removing oxidants from the skin so that it can resist environmental oxidative strain. Vitamin C collects in phagocytes, improving chemotaxis, phagocytosis, and eventually killing microorganisms. Numerous scientific studies and clinical trials describing potent immune-stimulating and antiviral effects have been published [18,19]. Vitamin C is required for apoptosis at the site of macrophage infection and removal of spent neutrophils; thereby, it reduces necrosis/endocytosis and possible tissue injury. However, it has been proven by various researchers that vitamin C can enhance the differentiation and multiplication of B cells and T cells [20,21], which may be due to its genetic regulation.

Vitamin C insufficiency can lead to declined immunity and increased chances of infections [22,23]. Then, due to the high rate of the inflammatory process and metabolic demands, infections can rigorously disturb vitamin C levels. The management of established infections needs considerably greater doses (grams) to be provided for the raised inflammatory reaction and metabolic demands [21].

#### 2.1.2 Vitamin E

Vitamin E is a group of four tocopherols (α-, β-, γ-, and δ-tocopherols) and four tocotrienols (α-, β-, γ-, and δ-tocotrienols) present in food. These types possess antioxidant action but cannot be converted into each other. Only α-tocopherol can meet the requirements of human vitamin E [24]. The primary dietetic source of vitamin E is plant oils, such as soybean, sunflower, corn, and walnut [25,26]. Oils have greater vitamin E content (vitamin E/100 g oil is about 50 mg or more). The ratio of α-, β-, γ-, and δ-tocopherol differs according to the type of the oil. For example, safflower oil has a high content of α-tocopherol, soybean and corn oil have the highest content of γ-tocopherol, and the ratio of α- and γ-tocopherol in cottonseed oil is similar. Vitamin E is also found in vegetable oils, nuts, seeds, fruits, and vegetables, including kale, spinach, pumpkin, red pepper, mango, and avocado [27].

Vitamin E is an effective fat-soluble antioxidant. Compared with other cells in the blood, it has a higher concentration in immune cells and is one of the best active nutrients to regulate immune function. Its deficiency has been revealed to disturb the usual function of the immune system and can be improved by supplementing vitamin E through foods. Vitamin E insufficiency is uncommon, and its supplementation beyond the recommended amount can improve the functioning of the immune system, e.g., decreases the risk of infection, specifically in the elderly [28].

The role of vitamin E in the immune system is usually connected with α-tocopherol. However, evolving indication suggests that other forms of vitamin E, containing other tocopherols and tocotrienols, may also have active immunomodulatory functions [29].

Alternative research disclosed that vitamin E is a fat-soluble antioxidant that can protect polyunsaturated fatty acids (PUFAs) in the membrane from oxidation, normalizing the manufacture of reactive oxygen species (ROS) and reactive nitrogen. Under normal and pathological conditions, the immunomodulatory effect of vitamin E has been found in both animal and human models. Due to the underestimation of the development, function, and regulation of dendritic cells, macrophages, natural killer (NK) cells, T cells, and B cells, recent research has focused on the role of vitamin E in specific immune cells. Vitamin E enhances cells to recognize the immune regulation mechanism [30].

In clinical, pre-clinical, and cell-based intervention studies, the mechanisms that cause vitamin E to affect the immune system and inflammation have been explored [29,31,32]. Vitamin E directly affects the integrity of T-cell membranes, signal transfer, and cell division and indirectly disturbs the inflammatory mediators produced by other immune cells, thus modifying the function of T cells. Vitamin E has medical importance in regulating immune function because it cannot only disturb the host’s vulnerability to infectious diseases (such as respiratory infections) that are susceptible to diseases (such as asthma) [33].

#### 2.1.3 Vitamin A

Vitamin A is a micronutrient that maintains eyesight, promotes growth and development, and protects the reliability of the human epithelium and mucus. Due to its important role in enhancing immune function, it is known as an anti-inflammatory vitamin. This vitamin participates in the development of the immune system and plays a supervisory role in the cellular immune response and humoral immune process. Vitamin A has a proven therapeutic effect on various infectious diseases [34]. Plant sources that contain vitamin A include carrots, pumpkins, papaya, and mangoes. Its deficiency can disturb innate immunity by inhibiting the usual restoration of the mucosal barrier caused by infection and weakening the function of neutrophils, macrophages, and NK cells. Vitamin A is also essential for adaptive immunity and plays a role in the growth of T-type helper (Th) B cells, and its possible deficiency decreases the antibody-mediated response directed by Th2 cells. Th1-mediated immunity is also reduced. These alterations in mucosal epithelial redevelopment and immune function may be responsible for the greater death rate of infants, young children, and pregnant women who have vitamin A deficiency [35].

In the case of people with low vitamin A content in their diet, contagious diseases can aggravate vitamin A deficit by decreasing intake, absorption, and increasing excretion. Contagious diseases that induce acute-phase responses can also change the assessment of vitamin A status by temporarily decreasing the serum retinol concentration [36].

#### 2.1.4 Vitamin B_6_

Vitamin B_6_ contains six pyridine derivatives [37] pyridoxal, pyridoxine, pyridoxamine, and their respective 5′-phosphates. It is a cofactor for certain enzymes, supporting them in performing their respective functions [38]. Plant-based foods, such as oats, bananas, wheat germ, soya beans, and peanuts, are good sources of B_6_ [39]. It helps control the isotype and the level of cysteine in the body. The human body needs B_6_ to absorb vitamin B_12_ and for the production of red blood cells and immune system cells [40]. Vitamin B_6_ can help the body produce a variety of neurotransmitters, such as serotonin, which transmit signals from one nerve cell to another. Serotonin is formed only from tryptophan. This transformation (tryptophan to serotonin) arises in the existence of pyridine phosphate, which is a derivative of vitamin B_6_ [41]. Vitamin B_6_ is essential for usual brain growth and function [42]. Pyridoxal 5′-phosphate is essential for the synthesis of the neurotransmitters serotonin, norepinephrine, epinephrine, and γ-aminobutyrate and, as such, is involved in both stimulating and inhibiting neurons [39,43]. It can help the body produce norepinephrine that affects mood and melatonin. Indications of severe deficit include muscular weakness, anxiety, irritability, sadness, failure to concentrate, and occasionally short-term memory loss. The recommended daily consumption of vitamin B_6_ for adults should be 1.3 mg [39]. Vitamin B_6_ aids in increasing the immune response to greater antibodies and aids the communication among cytokines and chemokines [44]. Its deficit decreases lymphocyte growth and multiplication, the production of antibodies, and the active cells of T [45].

#### 2.1.5 Vitamin B_9_

Folate is the natural form of vitamin B_9_, water-soluble and naturally found in many foods. It is also added to foods and sold as a supplement in the form of folic acid; this form is better absorbed than that from food sources. Folate helps to form DNA and RNA and is involved in protein metabolism. Leafy vegetables (including romaine lettuce, turnip greens, asparagus, spinach, broccoli, and Brussels sprouts), peanuts, whole grains, beans, fresh fruits, and sunflower seeds are the sources of vitamin B_9_. Vitamin B_9_ can also boost immunity. It usually plays a vital role in the biosynthesis of nucleic acids, proteins [46], RBCs, and nerve tissue [47]. Folic acid is important for the usual functioning of the brain and plays a main role in mental health. The lack of vitamin B_9_ can cause depression, insomnia, fatigue, and anxiety [48].

### 2.2 Minerals

#### 2.2.1 Zinc

Zinc (Zn) is an important trace mineral and plays a vital role in many physiological functions. Biologically active zinc is a bivalent cation mainly associated with enzymes and other proteins [49]. Rich plant-based food sources of Zn are legumes, including lentils and chickpeas, beans, nuts, and whole grains [50]. One of the significant functions of Zn is its effect on the immune system. It is essential for the growth and function of immune cells in the innate and adaptive immune system. Zn homeostasis is well controlled in cells, and any stress-free regulation will result in compromised normal function. Its insufficiency will adversely affect the hematopoietic function and affect the immune response at multiple molecular, cellular, and systemic levels. Zn contributes to the immune system and how alterations in intracellular zinc levels affect the immune response [51]. Zn affects numerous features of the immune system, from the skin barrier to the gene regulation of lymphocytes [52,53]. It is important for the usual growth and function of cells. Zn deficiency prevents T overgrowth of lymphocytes and certain functions (such as stimulation, Th1 cytokine production, and B lymphocyte aid) from affecting the development of adaptive immunity, such as the development of B lymphocytes and antibody production, especially globulin G immunity damaged [54]. Macrophages are key cells in many immune functions and are undesirably affected by zinc deficiency. Zn insufficiency can disturb intracellular destruction, cytokine production, and phagocytosis. Zn is the immune mediator that stems from the several roles of zinc in basic cell roles (such as DNA replication, RNA transcription, and cell division). Its insufficiency increases cell apoptosis [55].

A previous study showed that zinc is also an antioxidant and has anti-inflammatory effects. The beneficial effects of Zn have been reported in patients with acute infantile diarrhea, inflammatory bowel disease, prevention of age-related macular degeneration, and treatment of colds with Zn. In HL-60 cells (promyelocytic leukemia cell line), Zn improves the upregulation of A20 mRNA and decreases the triggering of NF-κB through the TRAF pathway, causing reduced gene expression of tumor necrosis factor α, interleukin (IL)-1β, and IL-8. It is reported that the addition of zinc diminishes the formation of oxidative stress markers and inflammatory cytokines in young and elderly people [56]. Zn acts as a modulator of immune reaction through its accessibility. When this mechanism is disturbed, the utilization of Zn will reduce, thus altering the existence, multiplication, and variation of cells in various organs and systems, especially immune system cells [57].

#### 2.2.2 Copper

Copper (Cu) is a trace mineral that plays an important role in humans [58]. Whole grains, beans, and nuts are good sources of Cu. Dark green leafy vegetables in the diet, plums, cocoa, black pepper, and dried fruits are also sources of Cu [59,60,61].

A link between Cu and innate immune function has been observed. Literature indicates that mild copper insufficiency in humans and animals is usually described as neutropenia [62]. This condition is partly linked to the reduction in the number of circulating neutrophils. Thus, it has been suggested that Cu is closely related to the differentiation, maturation, and proliferation of leukocytes [63]. Neutrophils are small and homogeneous cell populations, so they are probably to be an operational and valuable tool for evaluating human nutritional status [64]. As a result of Cu deficiency, isolated neutrophils showed declined phagocytic capability and/or decreased bactericidal action, but these factors were easily restored when Cu was added to the diet [65,66]. Some previous studies have shown that IL-2 Cu deficiency is reduced, which may be the mechanism of T-cell production. These outcomes indicate that even in the case of marginal deficiency, when the index total copper level is not affected by nutrition, proliferative response and the concentration of IL are decreased. In the case of severe Cu deficiency, the number of human peripheral blood neutrophils decreases, and as a consequence reduce their ability to produce superoxide anions and kill ingested microorganisms. When neutrophil-like HL-60 cells differentiate into more mature cell populations, they accumulate copper, and the increased activity of Cu/Zn superoxide dismutase or cytochrome C oxidase cannot reflect this accumulation. The identity of copper-binding proteins in these cell types can help understand new Cu functions.

#### 2.2.3 Selenium

Selenium (Se) is a micronutrient that exists in the soil and enters the food chain by incorporating plant protein [67]. However, some areas of the world (including the United Kingdom, New Zealand, and Northeast China) have low or insufficient Se content in the soil, which is related to the insufficient Se content in the human body. Although evident Se deficiency is rare, it is common in most immunocompromised patients [68]. Globally, 1–5 billion people suffer from Se insufficiency [69]. Se deficit is often in most immunocompromised patients, which may describe the greater susceptibility of these patients to viral and bacterial infections [51]. Se plays a vital role in the function of the immune system. This antioxidant contributes to decreasing oxidative stress in the body, thereby decreasing inflammation and enhancing immunity [70]. Research has shown that improved levels of selenium in the blood are associated with boosting immune responses [71]. Sufficient levels of Se are important for initiating immunity. Moreover, Se plays a role in regulating immune responses and chronic inflammation. Indication of the role of several selenoproteins in inflammation regulation and immunity has emerged, providing insights into the mechanism by which selenium affects these processes. It is associated with improving oxidative stress, but in the absence of selenium, other functions in immune cells (such as protein folding and calcium flux) are also affected. Supplementing adequate levels of Se in the diet can also affect the function of immune cells. In some cases, certain types of susceptibility and immunity can mainly affect selenium levels and sexual dimorphism [72,73]. There is a lot of evidence that a relevant intake of micronutrients (including Se) through diet or supplements can benefit health and immunity.

Se is a basic component of the amino acid selenocysteine incorporated into selenoproteins. Glutathione peroxidase and thioredoxin reductase (TrxR) are examples of selenoproteins with antioxidant functions. These proteins affect oxidative stress and therefore play a main role in modifying the immune system [74]. Se is an effective antioxidant that exerts natural effects by incorporating it into selenoproteins, which play a relevant role in the regulation of ROS and redox states in practically all tissues. Thus, selenium intake from diet strongly affects susceptibility and immune responses [75,76,77,78].

### 2.3 Phenolic compounds

Polyphenols are pharmacologically active components with immunomodulatory activity [79]. This category contains flavonoids and phenolic acids, which are ubiquitous in plants and exist as free aglycones or esterified forms with glucose and other carbohydrates (glycosides) [80]. Thus, the absorbed polyphenols interact with the intestinal immune system, leading to a protective response for the host [81]. Current evidence strongly suggests that polyphenols help avoid a variety of immune diseases, i.e., the polyphenols in red wine can considerably raise the level of IL-21 and decrease the release of IL-1β and IL-6 [82]. It is well known that many pathogens of human diseases are involved in immune function. This observation has led to extensive investigational research on immune mechanisms in many disease settings. Immune dysfunction has many unforeseen consequences. For instance, immune dysfunction of the intestinal mucosa can be the reason for the host’s diarrhea and may have an adverse impact on the balance of intestinal flora [83]. Hence, functional foods defined by providing specific nutrition or targeting multiple functional ingredients are considered a form of preventive medicine [84]. Polyphenols are biologically active substances that can stimulate intestinal health through several mechanisms, such as regulating immunity and mucosal inflammation. The innate immune system of the intestine holds three lines of defense: mucus layer, epithelium, and lamina propria. The mucus layer is the host’s first line of defense against foreign pathogens [85].

Flavonoids, including about 6,000 phenolic compounds, are the products of the secondary metabolism of plants. Flavonoids can be divided into flavonols (such as quercetin, kaempferol, and isoquercetin), flavones (such as luteolin, flavone glycosides, and apigenin), isoflavones (such as genistein and daidzein), flavanones (such as naringenin and hesperidin), flavanol (such as epicatechin, catechin, gallocatechin, and epigallocatechin), esters, and polymerized or condensed tannins and anthocyanins found in cocoa and tea (such as pelargonidin, anthocyanins, and malvidin found in red wine and berries) [86,87,88]. Flavonoids are often powerful plant pigments that protect free radical damage and support cells and cytokines that regulate immune responses [89]. Flavonoids play a crucial role in defending the immune system against respiratory infections [88,90]. The stability of polyphenols is different, particularly in the environment of intestinal digestion. For example, compounds, such as anthocyanins (flavonoids), in the duodenum are comparatively unstable [91], while the total polyphenols and anthocyanins in the simulated gastrointestinal digestion process are usually very stable, and the recovery rate is about 93–99% [92].

The flavonoids are in many foods, such as fruits and vegetables [93,94,95,96], nuts (walnuts and legumes) [97,98,99,100], spices, and drinks (red wine and tea) [101,102]. Chemically, flavonoids have a polyphenol structure, which gives them antioxidant activity. Though some of them are associated with these capacities, the biological activity of flavonoids goes beyond the properties of antioxidants. Certain types of flavonoids are effective against cancer [103,104], cardiovascular diseases (CVDs) [105,106,107], gastrointestinal changes [108,109], and related neurological syndromes, such as depression [110], epilepsy [111], and neurodegenerative diseases, i.e., AD [112,113].

## 3 Use of nutraceuticals in plant-based food against acute and chronic diseases

Currently, plant-based diets, vegetarianism, and veganism are gaining popularity. A properly composed plant diet provides all the necessary nutrients but can also have a positive effect on health [114]. Food of plant origin is credited with a positive influence in the prevention and treatment of many diseases. Table 2 presents a summary of the activity of some of the plant products.

**Table 2:** *In vitro* and *in vivo* trials of plant-based food against different diseases

Sources	Common or scientific names	Bioactive/functional components	Type of study	Disorders	Improvement	References
Vegetables	*Morinda officinalis*	Fructooligosaccharide	*In vivo*	ADs	Significantly influence the brain function	[193]
	Bok choy	Flavonols	*In vitro*	Colon cancer	Inhibit the cell proliferation of the human colon adenocarcinoma cell line	[194]
	Spinach	Glycolipids	*In vitro*	Cancer	Significantly affect human cancer cell proliferation	[195]
	Carrot	Polyacetylenic oxylipins falcarinol and falcarindiol	*In vitro*	Colorectal cancer	Significantly reduce the risk of colorectal cancer	[196]
	Potato	Peptide	*In vivo*	Kidney failure	Significantly reduce in the number of apoptotic cells	[197]
	Red cabbage	Phenolic acids and flavonoids	*In vivo*	CVDs	Significantly reduce the levels of cholesterol, triglycerides, and lipoproteins	[198]
Fruits	Apple	Phenolics and flavonoids	*In vivo*	Mammary tumors	Significantly prevent mammary cancer	[199]
	Banana peel	Phenols	*In vivo*	Acute liver failure	Significant decrease in liver function, total cholesterol, triglycerides, low-density lipoprotein, and very-low-density lipoprotein	[200]
	Mango	Gallic acid and gallotannins	*In vivo*	Inflammation diseases	Significantly reduce the inflammatory response	[201]
	Pomegranate and their peel	Non-enzymatic and enzymatic antioxidant molecules	*In vivo*	Human fertility	The amount of male sex hormones, such as testosterone, follicular stimulating hormone, and luteinizing hormone, was increased with an increase in the level of pomegranate and their peel	[202]
	Grapefruits	Limonin and naringin	*In vivo*	Osteoporosis	Reduce the calcium loss and higher plasma IGF-I level in rats fed with grapefruits	[203]
	Guava-strawberry, guava-blackberry, guava-soursop	Phenolic acids and flavonoids	*In vivo*	Hyperglycemic and hypercholesterolemic	Reduce the levels of plasma glucose, urea creatinine, total cholesterol, and triacylglycerol levels in rats fed with functional puree	[204]
Seeds	Soybean	Isoflavone	*In vivo*	Cardiovascular	Soybean diet significantly reduced the serum LDL cholesterol level	[205]
	Sesame seeds	Sesamin and sesamolin	*In vivo*	Liver diseases	The rats fed with sesame seeds revealed that the activity of enzymes involved in fatty acid synthesis, including fatty acid synthase, glucose-6-phosphate dehydrogenase, ATP-citrate lyase, and pyruvate kinase, was significantly reduced	[206]
	Chia seeds	Polyphenols and ALA	*In vivo*	Obesity	The activity of plasma increased in the formulated diet while lowering the activity of thiol levels, plasma catalase, and glutathione peroxidase	[207]
	Flaxseed	α-Linolenic acid	*In vivo*	CVD diabetes	Administration of flaxseed improves the lipid profile and reduces the LDL cholesterol level	[208]
	Pumpkin seed	Phenolic compounds, tocopherol isomers, and phytosterols	*In vivo*	Cancer arthritis	Induce cell cycle arrest, reduce inflammation, and improve magnesium absorption, which results in good health of bones along with regulated sugar level	[209]
Nuts	Almond	Vitamins, unsaturated fats, and minerals	*In vivo*	Lead toxicity	Increased the growth and nutritional consumption by reducing the appetite depressant effect of lead on gastrointestinal tract	[210]
	Hazelnut	Monounsaturated fatty acids and PUFA	*In vitro*	Nonalcoholic fatty liver hyperlipidemia	Diet contains different doses of hazelnut oil that affects the serum cholesterol profile and induced a high level of HDL	[211]
	Cashew nuts	Phenolic acid and flavonoids	*In vivo*	Gall stone anemia	Increased the formation of red blood cell by the administration of iron, improved the ability of carrying oxygen to the other tissues	[212]
Spices and herbs	Bay leaf	Essential oils	*In vivo*	Diabetes	Extract of leave (*L. nobilis*) has significant impact on blood glucose level	[213]
	Asafetida	Asafoetida	*In vivo*	Chronic and acute pain	Asafetida exhibited a significant antinociceptive impact on chronic and acute pain rats	[214]
	Black cumin	Cuminaldehyde, cymene, and terpenoids	*In vivo*	Renal toxicity	Significantly increased the minerals, vitamin D, nutritional markers, and antioxidant enzymes in rats fed with black cumin seed powder	[215]

### 3.1 Asthma attack and bronchitis

A diet of fresh fruits and vegetables can have a significant impact on the health of people with asthma. Foods of animal origin are rich in vitamin D, whereas vegetables are rich in β-carotene (carrots and leafy green vegetables). Moreover, food is a good source of magnesium, for example, spinach and pumpkin seeds [115]. Fruits and vegetables are also significant sources of antioxidants, such as beta-carotene and vitamins E and C. These substances aid in stopping particles called “free radicals,” which led to inflammation. Nutrients can improve the reaction of the immune system (the body’s resistance to bacteria) and can reduce inflammation in the respiratory tract [116,117]. Low vitamin D levels can cause more asthma attacks [118,119]. Rich in vitamin E plant-based diet is particularly beneficial for asthma [120]. Raw almonds, hazelnuts, seeds, and cruciferous vegetables (such as broccoli and kale) are good sources of this vitamin. Vitamin E can help reduce coughing and wheezing caused by asthma [121]. Investigations on asthma and diet have shown that undernourished young people are bound to have more frequent asthma symptoms. Lung function has also been proven to be worse in people who do not eat food with sufficient levels of vitamins C and E and ω-3 unsaturated fatty acids [122,123].

### 3.2 Broken bone

A bone break is an ailment where a ceaseless crack of bones happens. Likewise, breaks can be caused by certain ailments that debilitate bones [124], such as osteoporosis, certain malignancies, or osteogenesis imperfecta [125]. Bone tissue is principally an extracellular framework, not living cells. Consequently, open breaks and osteotomies require cautious protection methods and preventive utilization of anti-toxins [126]. An appropriate diet for healthy bones provides products containing vegetables, whole-meal products, nuts, and seeds. Calcium is found in green vegetables, dried beans, dark belt molasses, sesame seeds, and almonds [127]. Other nutrients are additionally significant for bone good condition, such as vitamin D, magnesium, vitamin B_12_, vitamin C, and vitamin K [128].

### 3.3 Common cold and flu

The basic virus is a viral disease of the nose and throat (upper respiratory parcel). The normal chilly, otherwise called the regular cold, is a viral contamination of the upper respiratory parcel, which predominantly influences the respiratory mucosa of the nose, throat, sinuses, and larynx [129]. Signs and side effects under 2 days after the infection contamination may show indications, such as sore throat, runny nose, sniffling, and fever [130,131].

Vitamins C and D have the most noteworthy advantage for patients and can decrease the danger of pneumonia. Different enhancements, such as echinacea, vitamin E, and zinc, have some clinical information [132]. Zinc represses viral replication and has been used in preliminary efforts for the treatment of the basic virus. The past survey recognized 18 randomized controlled preliminaries that enrolled 1,781 members, everything being equal, and contrasted zinc and a fake treatment (without zinc) [133]. It has been demonstrated that taking zinc within 24 h of the beginning of side effects can diminish the span of the regular cold [134].

### 3.4 Pneumonia and respiratory infection

Pneumonia is an infection that inflames the air sacs in one or both lungs. It is practically difficult to stay away from infections and microorganisms. However, certain dangerous components can build the opportunity of getting intense respiratory contamination [135]. A high-protein diet is beneficial to patients with pneumonia. Food sources, such as nuts, seeds, and beans, have mitigating properties. In addition, they fix harmed tissues and construct new tissues in the body. Green vegetables, such as kale, lettuce, and spinach, comprise abundant in supplements that can help treat this respiratory contamination. They contain cell reinforcements, which shield the body from irresistible substances. Citruses are plentiful in vitamin C, such as oranges, berries, and kiwis, which help fortify the safe framework, accordingly advancing quick recovery. Likewise, they contain cell reinforcements, which protect the human body [136].

### 3.5 Coronavirus disease (COVID-19)

COVID-19 is a group of infections that cause human respiratory sicknesses. Extreme acute respiratory syndrome, middle east respiratory syndrome, and the regular virus are COVID-19. Elderly people and individuals with hidden clinical issues, such as cardiovascular infection, diabetes, constant respiratory illnesses, and malignancy, are predisposed to COVID-19 [137]. Food sources of plant origin increase the number of beneficial microbes. By utilizing a lot of water, minerals (such as magnesium, selenium, or zinc), micronutrients, spices, food sources plentiful in vitamins C, D, and E, and a healthy lifestyle, individuals can defeat this disease [12,138,139]. Numerous investigations have shown that the amazing cell reinforcement glutathione and the bioflavonoid quercetin can prevent diseases, including COVID-19. Thus, plant-based food varieties assume an imperative part in improving the resistance [140].

### 3.6 Diabetes

It is commonly known that eating plant-derived substances from botanical and organic foods, such as legumes, whole grains, fruits, vegetables, nuts, [141] and natural products are advantageous treatments against type 2 diabetes [142]. Plant-based diets are eating patterns that highlight legumes, whole grains, vegetables, fruits, nuts, and seeds and depress nearly all livestock products [143]. A group of studies greatly subsidize the function of vegetarian diets and foods and their nutritional substances in lowering the chances of type 2 diabetes [144]. Confirmation from interposing and experimental studies revealed the assistance of plant-derived foods in healing macro- and microcirculatory problems and type 2 diabetes. Ideal nutrients to block and control type 2 diabetes are arguable. Although authentication recommends that the class and origin of carbohydrates (unprocessed versus processed), lipids (unsaturated versus saturated and cis-trans), and proteins (herbal versus animal) serve a vital purpose in the elimination and handling of type 2 diabetes. Plant-based diets are critical and have a variety of prospective mechanisms in the improvement of the endurance of insulin, furthermore the of fit bodyweight, increment in fiber and plant-based nutrients, food-micro-floral relations, and in lowering hydrogenated fat, progressive glycation end results, nitrosamines, and cofactor of iron [145].

Less processed and agricultural-based foods have better opposition and glycemic management by presenting multiple procedures [146]. Herbal foods are rich in soluble fiber, phytochemicals, and magnesium, all of which encourage insulin sensitivity [147]. Antioxidants like polyphenols can retard glucose intake, restore insulin production, minimize hepatic glucose yield, and upgrade glucose absorption [148]. Fiber, which is present mostly in plant foods, harmonizes post-meal glucose reaction and is provoked by probiotics to generate short-chain fatty acids that may enhance glucose feedback, motioning of insulin, and insulin reactivity [149]. Moreover, the dwindling impedance of insulin is linked with foods that aid in satisfaction, weight reduction, are rich in fibers, and are less energy dense [150]. Soluble fibers boost insulin response and eliminate swelling [151]. Ultimately, a meal rich in herbal foods and less in protein portion is more influential in digestion by accelerating good microbial species and declining the assembly of trimethylamine *N*-oxide, a composite bonded to insulin strength [152].

In the long run, vegetable origin proved to prevent weight reduction and obesity [153], which are defensive characteristics in the insulin fight, compared meat consumption that trigger gradually increasing weight. Likewise, when less caloric and more proteinaceous food is taken for weight reduction, it will nullify to support slimness [154]. Smith et al. [155] found that for overweight and menopausal women, a simple, protein-rich diet terminated the remedial consequences in the reduction of weight loss on voluntary muscles, and insulin impulsiveness is possible because of the magnified oxidative burden.

### 3.7 CVD

Plant-based diets come with various flavors, but they all promote ingredients linked to heart health, such as whole cereals, bananas, tomatoes, lentils, almonds, and olive oil [156]. These foods are high in fiber, vitamins, and minerals [157], tend to lower blood pressure and LDL cholesterol, minimize the risk of diabetes, and maintain a healthy weight, both of which reduce the risk of CVD [158]. Flavonoids obtained from dietary plant consumption support vascular endothelial cells, probably as antioxidants that inhibit low-density lipoprotein oxidation [159]. Research has linked that plant-based diets are reduced the risk of cardiovascular outcomes, especially whole grains, fruits, and berries [160]. According to the evidence from prospective cohort research, eating many plant-based foods (including fruits and vegetables, almonds, and whole grains) reduces the risk of coronary heart disease and stroke. Besides, mono-unsaturated fatty acids and PUFAs, ω3 fatty acids, antioxidant vitamins, minerals, and phytochemicals are supreme substances in CVD [161].

### 3.8 AD

Numerous epidemiological studies propose a connection between lifestyle, diet, AD, and different types of dementia. Besides, it has been demonstrated that metabolic conditions and issues, such as insulin opposition, weight, cardiovascular illnesses, diabetes, and AD, are unequivocally related. Dietary mediations and other preventive procedures can be powerful ways to deal with stopping or postponing the danger of AD, intellectual decay, and other non-mental comorbidities [162]. Numerous supplements take part in biochemical responses; for instance, admission of an eating regimen abundant in probiotics, cell reinforcements, plant-based food varieties, ω3 polyunsaturated unsaturated fats, soybeans, and nuts can help moderate AD. Besides, less utilization of creature-determined proteins, refined sugars, and low admission of saturated fats can be helpful in such a manner. Furthermore, plant-derived polyphenols are seen as important compounds that can help reduce the effects of AD [112].

### 3.9 Cancer

Botanical foodstuffs, such as fruits, vegetables, legumes, nuts, and cereals, are good sources of nutrients. Because this food is composed of some phenolic compounds and antioxidants, it reduces the risk of oxidative stress and saves cells from an injury that creates diseases like cancer. By promoting fiber utilization, plant foods can block the production of abnormal cells [163]. Moreover, fiber has been proven to protect against colorectal carcinoma [164]. Scientific studies have shown that whole grains, vegetables, fruits, beans, and nuts contain supplements and phytocomponents and exhibit the capability of altering gene expression [165]. Earlier studies exhibited that the most widely considered thing was the consequences of botanical food and nutrition on breast, colorectal, and gastrointestinal tumors. Organic and botanical diets exhibited remarkable protection against tumors and in addition to other cancers, long-lasting illnesses and disorders [166].

## 4 Conclusion

In this review article, a great deal of effort has been made to provide an understanding of how plant-based foods can be used in improving the immunity against acute and chronic diseases. Recently, it has been reported that plant-based foods are needed to maintain appropriate human body conditions. These foods mainly consist of fruits, vegetables, legumes, lentils, and beans. Functional components are present in leaves, roots, seeds, and grains and play significant roles in human health. The immune system can boost some plant-derived components, such as vitamins, minerals, antioxidants, and phenolic compounds, because these components aid in forming T cells, B cells, and antibodies and enhance the function of selenoproteins and neutrophils. A strong immune system can protect against acute diseases, such as viral diseases (COVID-19, pneumonia, common cold, and flu) and bacterial diseases (asthma and bronchitis). Chronic diseases, including cancer, diabetes, CVD, and AD risk, can be reduced by consuming plant-based foods because they are composed of components that inhibit oxidation and reduce blood sugar and cholesterol levels.

## References

[1] Key TJ, Appleby PN, Rosell MS. Health effects of vegetarian and vegan diets. Proc Nutr Soc. 2006;65(1):35–41. 10.1079/PNS2005481. 16441942

[2] Ströhle A, Waldmann A, Wolters M, Hahn A. Vegetarische ernährung: präventives potenzial und mögliche risiken. Wien Klin Wochenschr. 2006;118(19–20):580–93. 10.1007/s00508-006-0706-y. 17136332

[3] Craig WJ. Health effects of vegan diets. Am J Clin Nutr. 2009;89(5):1627S–33S. 10.3945/ajcn.2009.26736N. 19279075

[4] Rzymski P, Kulus M, Jankowski M, Dompe C, Bryl R, Petitte JN, et al. COVID-19 pandemic is a call to search for alternative protein sources as food and feed: A review of possibilities. Nutrients. 2021;13(1):150. 10.3390/nu13010150. PMC7830574 33466241

[5] Mishra S, Xu J, Agarwal U, Gonzales J, Levin S, Barnard ND. A multicenter randomized controlled trial of a plant-based nutrition program to reduce body weight and cardiovascular risk in the corporate setting: the GEICO study. Eur J Clin Nutr. 2013;67(7):718–24. 10.1038/ejcn.2013.92. PMC3701293 23695207

[6] Le L, Sabaté J. Beyond meatless, the health effects of vegan diets: Findings from the adventist cohorts. Nutrients. 2014;6(6):2131–47. 10.3390/nu6062131. PMC4073139 24871675

[7] McManus KD What is a plant-based diet and why should you try it? https://www.health.harvard.edu/blog/what-is-a-plant-based-diet-and-why-should-you-try-it-2018092614760. Published 2018. Accessed April 7, 2022.

[8] Francenia Santos-Sánchez N, Salas-Coronado R, Hernández-Carlos B, Villanueva-Cañongo C. Shikimic acid pathway in biosynthesis of phenolic compounds. In: Soto-Hernández M, ed. Plant Physiological Aspects of Phenolic Compounds. London, UK: IntechOpen; 2019. 10.5772/intechopen.83815.

[9] Singh JP, Kaur A, Shevkani K, Singh N, Singh B. Physicochemical characterisation of corn extrudates prepared with varying levels of beetroot (Beta vulgaris) at different extrusion temperatures. Int J Food Sci Technol. 2016;51(4):911–9. 10.1111/ijfs.13051.

[10] Wang A, Islam MN, Qin X, Wang H, Peng Y, Ma C. Purification, identification, and characterization of d-galactose-6-sulfurylase from marine algae (Betaphycus gelatinus). Carbohydr Res. 2014;388:94–9. 10.1016/j.carres.2013.12.010. 24632215

[11] Zhang B, Deng Z, Ramdath DD, Tang Y, Chen PX, Liu R, et al. Phenolic profiles of 20 Canadian lentil cultivars and their contribution to antioxidant activity and inhibitory effects on α-glucosidase and pancreatic lipase. Food Chem. 2015;172:862–72. 10.1016/j.foodchem.2014.09.144. 25442631

[12] Arshad MS, Khan U, Sadiq A, Khalid W, Hussain M, Yasmeen A, et al. Coronavirus disease (COVID‐19) and immunity booster green foods: A mini review. Food Sci Nutr. 2020;8(8):3971–6. 10.1002/fsn3.1719. PMC7300634 32837716

[13] Singh B, Singh JP, Kaur A, Singh N. Bioactive compounds in banana and their associated health benefits – A review. Food Chem. 2016;206:1–11. 10.1016/j.foodchem.2016.03.033. 27041291

[14] Rus H, Cudrici C, Niculescu F. The Role of the complement system in innate immunity. Immunol Res. 2005;33(2):103–12. 10.1385/IR:33:2:103. 16234578

[15] Barnard N, Goldman D, Loomis J, Kahleova H, Levin S, Neabore S, et al. Plant-based diets for cardiovascular safety and performance in endurance sports. Nutrients. 2019;11(1):130. 10.3390/nu11010130. PMC6356661 30634559

[16] García-Closas R, Berenguer A, Tormo MJ, Sánchez MJ, Quirós JR, Navarro C, et al. Dietary sources of vitamin C, vitamin E and specific carotenoids in Spain. Br J Nutr. 2004;91(6):1005–11. 10.1079/BJN20041130. 15182404

[17] Xu W, Islam MN, Cao X, Tian J, Zhu G. Effect of relative humidity on drying characteristics of microwave assisted hot air drying and qualities of dried finger citron slices. LWT. 2021;137:110413. 10.1016/j.lwt.2020.110413.

[18] Oudemans-van Straaten HM, Man AMS, de Waard MC. Vitamin C revisited. Crit Care. 2014;18(4):460. 10.1186/s13054-014-0460-x. PMC4423646 25185110

[19] McNamara R, Deane AM, Anstey J, Bellomo R. Understanding the rationale for parenteral ascorbate (vitamin C) during an acute inflammatory reaction: a biochemical perspective. Crit Care Resusc. 2018;20(3):174–9. 10.1016/s0022-4804(02)00083-5. http://www.ncbi.nlm.nih.gov/pubmed/30153778. 30153778

[20] Manning J, Mitchell B, Appadurai DA, Shakya A, Pierce LJ, Wang H, et al. Vitamin C promotes maturation of T-cells. Antioxid Redox Signal. 2013;19(17):2054–67. 10.1089/ars.2012.4988. PMC3869442 23249337

[21] Carr A, Maggini S. Vitamin C and immune function. Nutrients. 2017;9(11):1211. 10.3390/nu9111211. PMC5707683 29099763

[22] Maggini S, Wenzlaff S, Hornig D. Essential role of vitamin c and zinc in child immunity and health. J Int Med Res. 2010;38(2):386–414. 10.1177/147323001003800203. 20515554

[23] Kim Y, Kim H, Bae S, Choi J, Lim SY, Lee N, et al. Vitamin C is an essential factor on the anti-viral immune responses through the production of interferon-α/β at the initial stage of influenza a virus (H3N2) infection. Immune Netw. 2013;13(2):70. 10.4110/in.2013.13.2.70. PMC3659258 23700397

[24] Traber MG. Vitamin E regulatory mechanisms. Annu Rev Nutr. 2007;27(1):347–62. 10.1146/annurev.nutr.27.061406.093819. 17439363

[25] Kmiecik D, Fedko M, Siger A, Kowalczewski PŁ. Nutritional quality and oxidative stability during thermal processing of cold-pressed oil blends with 5:1 ratio of ω6/ω3 fatty acids. Foods. 2022;11(8):1081. 10.3390/foods11081081. PMC9030854 35454668

[26] Gimeno E, Castellote A, Lamuela-Raventós R, de la Torre M, López-Sabater M. Rapid determination of vitamin E in vegetable oils by reversed-phase high-performance liquid chromatography. J Chromatogr A. 2000;881(1–2):251–4. 10.1016/S0021-9673(00)00219-3. 10905708

[27] Matthäus B, Musazcan Özcan M. Oil content, fatty acid composition and distributions of vitamin-E-active compounds of some fruit seed oils. Antioxidants. 2015;4(1):124–33. 10.3390/antiox4010124. PMC4665574 26785341

[28] Schneider C. Chemistry and biology of vitamin E. Mol Nutr Food Res. 2005;49(1):7–30. 10.1002/mnfr.200400049. 15580660

[29] Lewis ED, Meydani SN, Wu D. Regulatory role of vitamin E in the immune system and inflammation. IUBMB Life. 2019;71(4):487–94. 10.1002/iub.1976. PMC7011499 30501009

[30] Lee G, Han S. The role of vitamin E in immunity. Nutrients. 2018;10(11):1614. 10.3390/nu10111614. PMC6266234 30388871

[31] Wu D, Meydani SN. Age‐associated changes in immune and inflammatory responses: impact of vitamin E intervention. J Leukoc Biol. 2008;84(4):900–14. 10.1189/jlb.0108023. PMC2538592 18596135

[32] Wallert M, Börmel L, Lorkowski S. Inflammatory diseases and vitamin E – what do we know and where do we go? Mol Nutr Food Res. 2021;65(1):2000097. 10.1002/mnfr.202000097. 32692879

[33] Rizvi S, Raza ST, Siddiqi Z, Abbas S, Mahdi F. Association of angiotensin-converting enzyme and glutathione S-transferase gene polymorphisms with body mass index among hypertensive North Indians. Sultan Qaboos Univ Med J. 2015;15(4):e477–85. 10.18295/squmj.2015.15.04.006. PMC4664091 26629373

[34] Huang Z, Liu Y, Qi G, Brand D, Zheng S. Role of vitamin A in the immune system. J Clin Med. 2018;7(9):258. 10.3390/jcm7090258. PMC6162863 30200565

[35] Stephensen CB. Vitamin A, infection, and immune function. Annu Rev Nutr. 2001;21(1):167–92. 10.1146/annurev.nutr.21.1.167. 11375434

[36] Mora JR, Iwata M, von Andrian UH. Vitamin effects on the immune system: vitamins A and D take centre stage. Nat Rev Immunol. 2008;8(9):685–98. 10.1038/nri2378. PMC2906676 19172691

[37] Rosenberg IH. A History of the isolation and identification of vitamin B6. Ann Nutr Metab. 2012;61(3):236–8. 10.1159/000343113. 23183295

[38] Hellmann H, Mooney S. Vitamin B6: A molecule for human health? Molecules. 2010;15(1):442–59. 10.3390/molecules15010442. PMC6257116 20110903

[39] Stover PJ, Field MS. Vitamin B-6. Adv Nutr. 2015;6(1):132–3. 10.3945/an.113.005207. PMC4288272 25593152

[40] Lewicki S, Leśniak M, Bertrandt J, Kalicki B, Kubiak JZ, Lewicka A. The long-term effect of a protein-deficient-diet enriched with vitamin B6 on the blood parameters in unexercised and exercised rats. Food Agric Immunol. 2018;29(1):722–34. 10.1080/09540105.2018.1439900.

[41] Shabbir F, Patel A, Mattison C, Bose S, Krishnamohan R, Sweeney E, et al. Effect of diet on serotonergic neurotransmission in depression. Neurochem Int. 2013;62(3):324–9. 10.1016/j.neuint.2012.12.014. 23306210

[42] Blows WT. Neurotransmitters of the brain: Serotonin noradrenaline (Norepinephrine), and dopamine. J Neurosci Nurs. 2000;32(4):234–8. 10.1097/01376517-200008000-00008. 10994538

[43] Sato K. Why is vitamin B6 effective in alleviating the symptoms of autism? Med Hypotheses. 2018;115:103–6. 10.1016/j.mehy.2018.04.007. 29685187

[44] Kunisawa J, Kiyono H. Vitamin-mediated regulation of intestinal immunity. Front Immunol. 2013;4:189. 10.3389/fimmu.2013.00189. PMC3708512 23874335

[45] Rail LC, Meydani SN. Vitamin B6 and immune competence. Nutr Rev. 2009;51(8):217–25. 10.1111/j.1753-4887.1993.tb03109.x. 8302491

[46] Stover PJ. Physiology of folate and vitamin B 12 in health and disease. Nutr Rev. 2004;62:S3–S12. 10.1111/j.1753-4887.2004.tb00070.x. 15298442

[47] Huskisson E, Maggini S, Ruf M. The influence of micronutrients on cognitive function and performance. J Int Med Res. 2007;35(1):1–19. 10.1177/147323000703500101. 17408051

[48] Mielgo-Ayuso J, Aparicio-Ugarriza R, Olza J, Aranceta-Bartrina J, Gil Á, Ortega R, et al. Dietary intake and food sources of niacin, riboflavin, thiamin and vitamin B6 in a Representative sample of the spanish population. The ANIBES study. Nutrients. 2018;10(7):846. 10.3390/nu10070846. PMC6073544 29966236

[49] Jurowski K, Szewczyk B, Nowak G, Piekoszewski W. Biological consequences of zinc deficiency in the pathomechanisms of selected diseases. J Biol Inorg Chem. 2014;19(7):1069–79. 10.1007/s00775-014-1139-0. PMC4175048 24748223

[50] Saunders AV, Craig WJ, Baines SK. Zinc and vegetarian diets. Med J Aust. 2013;199(S4):S17–S21. 10.5694/mja11.11493. 25369924

[51] Gammoh NZ, Rink L. Zinc and the immune system. Nutrition and Immunity. Cham: Springer International Publishing; 2019. p. 127–58. 10.1007/978-3-030-16073-9_8.

[52] Hojyo S, Fukada T. Roles of zinc signaling in the immune system. J Immunol Res. 2016;2016:1–21. 10.1155/2016/6762343. PMC5107842 27872866

[53] Skrajnowska D, Bobrowska-Korczak B. Role of zinc in immune system and anti-cancer defense mechanisms. Nutrients. 2019;11(10):2273. 10.3390/nu11102273. PMC6835436 31546724

[54] Dardenne M. Zinc and immune function. Eur J Clin Nutr. 2002;56(S3):S20–3. 10.1038/sj.ejcn.1601479. 12142956

[55] Shankar AH, Prasad AS. Zinc and immune function: the biological basis of altered resistance to infection. Am J Clin Nutr. 1998;68(2):447S–63S. 10.1093/ajcn/68.2.447S. 9701160

[56] Prasad AS. Zinc in human health: Effect of zinc on immune cells. Mol Med. 2008;14(5–6):353–7. 10.2119/2008-00033.Prasad. PMC2277319 18385818

[57] Bonaventura P, Benedetti G, Albarède F, Miossec P. Zinc and its role in immunity and inflammation. Autoimmun Rev. 2015;14(4):277–85. 10.1016/j.autrev.2014.11.008. 25462582

[58] Konikowska K, Mandecka A. Trace elements in human nutrition. Recent Advances in Trace Elements. Chichester, UK: John Wiley & Sons, Ltd; 2018. p. 339–72. 10.1002/9781119133780.ch17.

[59] Gunturu S, Dharmarajan TS. Copper and zinc. Geriatric Gastroenterology. Cham: Springer International Publishing; 2020. p. 1–17. 10.1007/978-3-319-90761-1_25-1.

[60] Feitosa S, Greiner R, Meinhardt A-K, Müller A, Almeida D, Posten C. Effect of traditional household processes on iron, zinc and copper bioaccessibility in black bean (Phaseolus vulgaris L.). Foods. 2018;7(8):123. 10.3390/foods7080123. PMC6111528 30065167

[61] Cabrera C, Lloris F, Giménez R, Olalla M, López MC. Mineral content in legumes and nuts: contribution to the Spanish dietary intake. Sci Total Env. 2003;308(1–3):1–14. 10.1016/S0048-9697(02)00611-3. 12738197

[62] Zidar BL, Shadduck RK, Zeigler Z, Winkelstein A. Observations on the anemia and neutropenia of human copper deficiency. Am J Hematol. 1977;3(2):177–85. 10.1002/ajh.2830030209. 304669

[63] Percival SS. Copper and immunity. Am J Clin Nutr. 1998;67(5):1064S–8S. 10.1093/ajcn/67.5.1064S. 9587153

[64] Bonham M, O’Connor JM, Hannigan BM, Strain JJ. The immune system as a physiological indicator of marginal copper status? Br J Nutr. 2002;87(5):393–403. 10.1079/BJN2002558. 12010579

[65] Xin Z, Waterman DF, Hemken RW, Harmon RJ. Effects of copper status on neutrophil function, superoxide dismutase, and copper distribution in steers. J Dairy Sci. 1991;74(9):3078–85. 10.3168/jds.S0022-0302(91)78493-2. 1779061

[66] Djoko KY, Ong CY, Walker MJ, McEwan AG. The role of copper and zinc toxicity in innate immune defense against bacterial pathogens. J Biol Chem. 2015;290(31):18954–61. 10.1074/jbc.R115.647099. PMC4521016 26055706

[67] Shahid M, Niazi NK, Khalid S, Murtaza B, Bibi I, Rashid MI. A critical review of selenium biogeochemical behavior in soil-plant system with an inference to human health. Env Pollut. 2018;234:915–34. 10.1016/j.envpol.2017.12.019. 29253832

[68] Combs GF. Selenium in global food systems. Br J Nutr. 2001;85(5):517–47. 10.1079/BJN2000280. 11348568

[69] Gill H, Walker G. Selenium, immune function and resistance to viral infections. Nutr Diet. 2008;65:S41–7. 10.1111/j.1747-0080.2008.00260.x.

[70] Chan S, Gerson B, Subramaniam S. The role of copper, molybdenum, selenium, and zinc in nutrition and health. Clin Lab Med. 1998;18(4):673–85. 10.1016/S0272-2712(18)30143-4. 9891606

[71] Sordillo LM. Selenium-dependent regulation of oxidative stress and immunity in periparturient dairy cattle. Vet Med Int. 2013;2013:1–8. 10.1155/2013/154045. PMC3557619 23401850

[72] Duan S, Chen S, Liang W, Chen M, Chen Y, Guo M. Dietary selenium deficiency facilitated reduced stomatin and phosphatidylserine externalization, increasing erythrocyte osmotic fragility in mice. Biol Trace Elem Res. 2021;199(2):594–603. 10.1007/s12011-020-02162-3. 32328968

[73] Huang Z, Rose AH, Hoffmann PR. The role of selenium in inflammation and immunity: from molecular mechanisms to therapeutic opportunities. Antioxid Redox Signal. 2012;16(7):705–43. 10.1089/ars.2011.4145. PMC3277928 21955027

[74] Arthur JR, McKenzie RC, Beckett GJ. Selenium in the immune system. J Nutr. 2003;133(5):1457S–9S. 10.1093/jn/133.5.1457S. 12730442

[75] Kieliszek M, Bano I, Zare H. A comprehensive review on selenium and its effects on human health and distribution in middle eastern countries. Biol Trace Elem Res. 2022;200(3):971–87. 10.1007/s12011-021-02716-z. PMC8761138 33884538

[76] Kieliszek M. In: Advances in Food and Nutrition Research. Michael Eskin, ed. Cambridge, Massachusetts, USA: Academic Press; 2021;96:417–29. 10.1016/bs.afnr.2021.02.019.

[77] Kieliszek M, Błażejak S. Current knowledge on the importance of selenium in food for living organisms: A review. Molecules. 2016;21(5):609. 10.3390/molecules21050609. PMC6274134 27171069

[78] Hoffmann PR, Berry MJ. The influence of selenium on immune responses. Mol Nutr Food Res. 2008;52(11):1273–80. 10.1002/mnfr.200700330. PMC3723386 18384097

[79] Szliszka E, Krol W. The role of dietary polyphenols in tumor necrosis factor-related apoptosis inducing ligand (TRAIL)-induced apoptosis for cancer chemoprevention. Eur J Cancer Prev. 2011;20(1):63–9. 10.1097/CEJ.0b013e32833ecc48. 20861738

[80] Ma Y, Kosińska-Cagnazzo A, Kerr WL, Amarowicz R, Swanson RB, Pegg RB. Separation and characterization of soluble esterified and glycoside-bound phenolic compounds in dry-blanched peanut skins by liquid chromatography–electrospray ionization mass spectrometry. J Agric Food Chem. 2014;62(47):11488–504. 10.1021/jf503836n. 25354220

[81] Pandey KB, Rizvi SI. Plant polyphenols as dietary antioxidants in human health and disease. Oxid Med Cell Longev. 2009;2(5):270–8. 10.4161/oxim.2.5.9498. PMC2835915 20716914

[82] Magrone T, Jirillo E, Spagnoletta A, Magrone M, Russo MA, Fontana S, et al. Immune profile of obese people and in vitro effects of red grape polyphenols on peripheral blood mononuclear cells. Oxid Med Cell Longev. 2017;2017:1–11. 10.1155/2017/9210862. PMC5294383 28243360

[83] Williams AR, Krych L, Fauzan Ahmad H, Nejsum P, Skovgaard K, Nielsen DS, et al. A polyphenol-enriched diet and Ascaris suum infection modulate mucosal immune responses and gut microbiota composition in pigs. PLoS One. 2017;12(10):e0186546. 10.1371/journal.pone.0186546. PMC5640243 29028844

[84] del Cornò M, Scazzocchio B, Masella R, Gessani S. Regulation of dendritic cell function by dietary polyphenols. Crit Rev Food Sci Nutr. 2016;56(5):737–47. 10.1080/10408398.2012.713046. 24941314

[85] Xu X-R. Dysregulation of mucosal immune response in pathogenesis of inflammatory bowel disease. World J Gastroenterol. 2014;20(12):3255. 10.3748/wjg.v20.i12.3255. PMC3964397 24695798

[86] Jäger A, Saaby L. Flavonoids and the CNS. Molecules. 2011;16(2):1471–85. 10.3390/molecules16021471. PMC6259921 21311414

[87] Pérez-Cano F, Castell M. Flavonoids, inflammation and immune system. Nutrients. 2016;8(10):659. 10.3390/nu8100659. PMC5084045 27775647

[88] Somerville VS, Braakhuis AJ, Hopkins WG. Effect of flavonoids on upper respiratory tract infections and immune function: A systematic review and meta-analysis. Adv Nutr. 2016;7(3):488–97. 10.3945/an.115.010538. PMC4863266 27184276

[89] Hechtman L Clinical naturopathic medicine. Cambridge, MA: Elsevier Health Sciences; 2018.

[90] Liskova A, Samec M, Koklesova L, Samuel SM, Zhai K, Al-Ishaq RK, et al. Flavonoids against the SARS-CoV-2 induced inflammatory storm. Biomed Pharmacother. 2021;138:111430. 10.1016/j.biopha.2021.111430. PMC7906511 33662680

[91] Mosele JI, Macià A, Romero M-P, Motilva M-J, Rubió L. Application of in vitro gastrointestinal digestion and colonic fermentation models to pomegranate products (juice, pulp and peel extract) to study the stability and catabolism of phenolic compounds. J Funct Foods. 2015;14:529–40. 10.1016/j.jff.2015.02.026.

[92] Correa-Betanzo J, Allen-Vercoe E, McDonald J, Schroeter K, Corredig M, Paliyath G. Stability and biological activity of wild blueberry (Vaccinium angustifolium) polyphenols during simulated in vitro gastrointestinal digestion. Food Chem. 2014;165:522–31. 10.1016/j.foodchem.2014.05.135. 25038707

[93] Hertog MGL, Hollman PCH, Katan MB. Content of potentially anticarcinogenic flavonoids of 28 vegetables and 9 fruits commonly consumed in the Netherlands. J Agric Food Chem. 1992;40(12):2379–83. 10.1021/jf00024a011.

[94] Ahmed M, Eun J-B. Flavonoids in fruits and vegetables after thermal and nonthermal processing: A review. Crit Rev Food Sci Nutr. 2018;58(18):3159–88. 10.1080/10408398.2017.1353480. 29035571

[95] Núñez Sellés AJ, Agüero JA, Paz LN. GC-MS analysis of mango stem bark extracts (Mangifera indica L.), Haden variety. Possible contribution of volatile compounds to its health effects. Open Chem. 2021;19(1):27–38. 10.1515/chem-2021-0192.

[96] Noman OM, Nasr FA, Alqahtani AS, Al-zharani M, Cordero MAW, Alotaibi AA, et al. Comparative study of antioxidant and anticancer activities and HPTLC quantification of rutin in white radish (Raphanus sativus L.) leaves and root extracts grown in Saudi Arabia. Open Chem. 2021;19(1):408–16. 10.1515/chem-2021-0042.

[97] Tsantili E, Konstantinidis K, Christopoulos MV, Roussos PA. Total phenolics and flavonoids and total antioxidant capacity in pistachio (Pistachia vera L.) nuts in relation to cultivars and storage conditions. Sci Hortic (Amst). 2011;129(4):694–701. 10.1016/j.scienta.2011.05.020.

[98] Ashokkumar K, Pandian A, Murugan M, Dhanya MK, Sathyan T, Sivakumar P, et al. Profiling bioactive flavonoids and carotenoids in select south Indian spices and nuts. Nat Prod Res. 2020;34(9):1306–10. 10.1080/14786419.2018.1557179. 30672326

[99] Velazquez E R, Silva L, Peix A. Legumes: A healthy and ecological source of flavonoids. Curr Nutr Food Sci. 2010;6(2):109–44. 10.2174/157340110791233247.

[100] Bolling BW, Chen C-YO, McKay DL, Blumberg JB. Tree nut phytochemicals: Composition, antioxidant capacity, bioactivity, impact factors. A systematic review of almonds, Brazils, cashews, hazelnuts, macadamias, pecans, pine nuts, pistachios and walnuts. Nutr Res Rev. 2011;24(2):244–75. 10.1017/S095442241100014X. 22153059

[101] Wang H, Provan GJ, Helliwell K. Tea flavonoids: Their functions, utilisation and analysis. Trends Food Sci Technol. 2000;11(4–5):152–60. 10.1016/S0924-2244(00)00061-3.

[102] Cheynier V. Flavonoids: chemistry, biochemistry and applications. Flavonoids in Wine. Boca Raton, FL, USA: CRC Press LLC; 2006. p. 263–318.

[103] Fantini M, Benvenuto M, Masuelli L, Frajese G, Tresoldi I, Modesti A, et al. In vitro and in vivo antitumoral effects of combinations of polyphenols, or polyphenols and anticancer drugs: perspectives on cancer treatment. Int J Mol Sci. 2015;16(12):9236–82. 10.3390/ijms16059236. PMC4463587 25918934

[104] Hosein Farzaei M, Bahramsoltani R, Rahimi R. Phytochemicals as adjunctive with conventional anticancer therapies. Curr Pharm Des. 2016;22(27):4201–18. 10.2174/1381612822666160601100823. 27262332

[105] El Haouari M, Rosado JA. Medicinal plants with antiplatelet activity. Phyther Res. 2016;30(7):1059–71. 10.1002/ptr.5619. 27062716

[106] Sirtori CR, Arnoldi A, Cicero AFG. Nutraceuticals for blood pressure control. Ann Med. 2015;47(6):447–56. 10.3109/07853890.2015.1078905. 26362125

[107] Grassi D, Desideri G, Mai F, Martella L, De Feo M, Soddu D, et al. Cocoa, glucose tolerance, and insulin signaling: Cardiometabolic protection. J Agric Food Chem. 2015;63(45):9919–26. 10.1021/acs.jafc.5b00913. 26126077

[108] Jain P. Secondary metabolites for antiulcer activity. Nat Prod Res. 2016;30(6):640–56. 10.1080/14786419.2015.1036269. 25920371

[109] Diniz TC, Silva JC, Lima-Saraiva SRG, Ribeiro FPR, Pacheco AGM, de Freitas RM, et al. The role of flavonoids on oxidative stress in epilepsy. Oxid Med Cell Longev. 2015;2015:1–9. 10.1155/2015/171756. PMC4306219 25653736

[110] Guan L-P, Liu B-Y. Antidepressant-like effects and mechanisms of flavonoids and related analogues. Eur J Med Chem. 2016;121:47–57. 10.1016/j.ejmech.2016.05.026. 27214511

[111] Sucher NJ, Carles MC. A pharmacological basis of herbal medicines for epilepsy. Epilepsy Behav. 2015;52:308–18. 10.1016/j.yebeh.2015.05.012. 26074183

[112] Shabbir U, Rubab M, Tyagi A. Oh D-H. curcumin and its derivatives as theranostic agents in Alzheimer’s disease: The implication of nanotechnology. Int J Mol Sci. 2020;22(1):196. 10.3390/ijms22010196. PMC7795367 33375513

[113] Bell L, Lamport D, Butler L, Williams C. A review of the cognitive effects observed in humans following acute supplementation with flavonoids, and their associated mechanisms of action. Nutrients. 2015;7(12):10290–306. 10.3390/nu7125538. PMC4690090 26690214

[114] Pye A, Bash K, Joiner A, Beenstock J. Good for the planet and good for our health: the evidence for whole-food plant-based diets. BJPsych Int. March 2022;1–3. 10.1192/bji.2022.7. https://www.cambridge.org/core/journals/bjpsych-international/article/good-for-the-planet-and-good-for-our-health-the-evidence-for-wholefood-plantbased-diets/CFD0B67B9653F9A250224ABCA8FA65F6.

[115] Beausoleil JL, Fiedler J, Spergel JM. Food intolerance and childhood asthma. Pediatr Drugs. 2007;9(3):157–63. 10.2165/00148581-200709030-00004. 17523696

[116] Kaur P, Ghoshal G, Jain A. Bio-utilization of fruits and vegetables waste to produce β-carotene in solid-state fermentation: Characterization and antioxidant activity. Process Biochem. 2019;76:155–64. 10.1016/j.procbio.2018.10.007.

[117] Ravimannan N, Nisansala A. Study on antioxidant activity in fruits and vegetables – A Review. Int J Adv Res Biol Sci. 2017;4(3):93–101. 10.22192/ijarbs.2017.04.03.010.

[118] Kang Q, Zhang X, Liu S, Huang F. Correlation between the vitamin D levels and asthma attacks in children: Evaluation of the effects of combination therapy of atomization inhalation of budesonide, albuterol and vitamin D supplementation on asthmatic patients. Exp Ther Med. November 2018;15(1):727–32. 10.3892/etm.2017.5436. PMC5772657 29399078

[119] Forno E, Bacharier LB, Phipatanakul W, Guilbert TW, Cabana MD, Ross K, et al. Effect of vitamin D3 supplementation on severe asthma exacerbations in children with asthma and low vitamin D levels. JAMA. 2020;324(8):752. 10.1001/jama.2020.12384. PMC7448830 32840597

[120] Iikura M. Plant-based diets and asthma. In: Mariotti F, ed. Vegetarian and Plant-Based Diets in Health and Disease Prevention. Cambridge, Massachusetts: Academic Press; 2017. p. 483–91. 10.1016/B978-0-12-803968-7.00027-7.

[121] Sumayya S, Parveen S, Hussain MA, Nayak SS. Role of diet in asthma and chronic obstructive pulmonary disease. World J Biol Pharm Heal Sci. 2021;5(3):53–63. 10.30574/wjbphs.2021.5.3.0026.

[122] Oudah NAR, Al-Teea KSC, Mohammed AA. Using the level of serum YKL-40 as an indicator to the pathogenesis of allergic asthma and helminths infection. Indian J Public Heal Res Dev. 2019;10(10):2898. 10.5958/0976-5506.2019.03314.X.

[123] Omar S, Kothiyal P, Jauhari R. Nutrition & asthma-feature review. Pharma Innov. 2017;6(7):229.

[124] Ralston SH. Bone structure and metabolism. Med (Baltim). 2017;45(9):560–4. 10.1016/j.mpmed.2017.06.008.

[125] Sinaki M. Osteoporosis. In: Cifu DX, ed. Braddom’s Physical Medicine and Rehabilitation. Cambridge, Massachusetts: Elsevier; 2021. p. 690–714.e3. 10.1016/B978-0-323-62539-5.00034-5.

[126] Koons GL, Diba M, Mikos AG. Materials design for bone-tissue engineering. Nat Rev Mater. 2020;5(8):584–603. 10.1038/s41578-020-0204-2.

[127] Weaver C. Nutrition and bone health. Oral Dis. 2017;23(4):412–5. 10.1111/odi.12515. 27250737

[128] Singh V. Medicinal plants and bone healing. Natl J Maxillofac Surg. 2017;8(1):4. 10.4103/0975-5950.208972. PMC5512407 28761270

[129] Allan GM, Arroll B. Prevention and treatment of the common cold: making sense of the evidence. Can Med Assoc J. 2014;186(3):190–9. 10.1503/cmaj.121442. PMC3928210 24468694

[130] Eccles R. Understanding the symptoms of the common cold and influenza. Lancet Infect Dis. 2005;5(11):718–25. 10.1016/S1473-3099(05)70270-X. PMC7185637 16253889

[131] Heikkinen T, Järvinen A. The common cold. Lancet. 2003;361(9351):51–9. 10.1016/S0140-6736(03)12162-9. PMC7112468 12517470

[132] Moyad MA. Conventional and alternative medical advice for cold and flu prevention: what should be recommended and what should be avoided? Urol Nurs 2009;29(6):455–8. http://www.ncbi.nlm.nih.gov/pubmed/20088240. 20088240

[133] Lin R-J, Huang C-H, Liu P-C, Lin I-C, Huang Y-L, Chen A-Y, et al. Zinc finger protein ZFP36L1 inhibits influenza A virus through translational repression by targeting HA, M and NS RNA transcripts. Nucleic Acids Res. June 2020;13:7371–84. 10.1093/nar/gkaa458. PMC7367194 32556261

[134] Joachimiak MP. Zinc against COVID-19? Symptom surveillance and deficiency risk groups. PLoS Negl Trop Dis. 2021;15(1):e0008895. 10.1371/journal.pntd.0008895. PMC7781367 33395417

[135] Edin A, Eilers H, Allard A. Evaluation of the biofire filmarray pneumonia panel plus for lower respiratory tract infections. Infect Dis (Auckl). 2020;52(7):479–88. 10.1080/23744235.2020.1755053. 32319831

[136] Xu D-P, Li Y, Meng X, Zhou T, Zhou Y, Zheng J, et al. Natural antioxidants in foods and medicinal plants: Extraction, assessment and resources. Int J Mol Sci. 2017;18(1):96. 10.3390/ijms18010096. PMC5297730 28067795

[137] Zimmermann P, Curtis N. Coronavirus infections in children including COVID-19. Pediatr Infect Dis J. 2020;39(5):355–68. 10.1097/INF.0000000000002660. PMC7158880 32310621

[138] Kieliszek M, Lipinski B. Selenium supplementation in the prevention of coronavirus infections (COVID-19. Med Hypotheses. 2020;143:109878. 10.1016/j.mehy.2020.109878. PMC7246001 32464491

[139] Kieliszek M. Selenium in the prevention of SARS-CoV-2 and other viruses. Biol Trace Elem Res. March 2022. https://link.springer.com/article/10.1007/s12011-022-03208-4. 10.1007/s12011-022-03208-4 PMC8934016 35305539

[140] GarciaS, Pandemics, Traditional Plant-Based Remedies. A historical-botanical review in the era of COVID19. Front Plant Sci. 2020;11:571042. 10.3389/fpls.2020.571042. PMC7485289 32983220

[141] Albuquerque TG, Nunes MA, Bessada SMF, Costa HS, Oliveira MBPP. Biologically active and health promoting food components of nuts, oilseeds, fruits, vegetables, cereals, and legumes. Chemical Analysis of Food. Cambridge, Massachusetts: Elsevier; 2020. p. 609–56. 10.1016/B978-0-12-813266-1.00014-0.

[142] Chettri P, Chandran SP. Role of dietary fibers in reducing the risk of type 2 diabetes. Int J Phys Educ Sport Heal. 2020;7(4):71–7. 10.22271/kheljournal.2020.v7.i4b.1772.

[143] Sawicka B, Umachandran K, El-Esawi MA. Plant-based nutrition supplementation on the well-being of servicemen. Phytochemistry, the Military and Health. Cambridge, Massachusetts: Elsevier; 2021. p. 377–404. 10.1016/B978-0-12-821556-2.00018-9.

[144] Pawlak R. Vegetarian diets in the prevention and management of diabetes and its complications. Diabetes Spectr. 2017;30(2):82–8. 10.2337/ds16-0057. PMC5439360 28588373

[145] McMacken M, Shah S. A plant-based diet for the prevention and treatment of type 2 diabetes. J Geriatr Cardiol. 2017;14:342–54. 10.11909/j.issn.1671-5411.2017.05.009. PMC5466941 28630614

[146] Parillo M, Riccardi G. Diet composition and the risk of type 2 diabetes: epidemiological and clinical evidence. Br J Nutr. 2004;92(1):7–19. 10.1079/BJN20041117. 15230984

[147] Alshali KZ. Review of herb supplement use in type 2 diabetes. Arch Pharm Pract. 2020;11(2):42–9.

[148] Cao H, Ou J, Chen L, Zhang Y, Szkudelski T, Delmas D, et al. Dietary polyphenols and type 2 diabetes: Human Study and Clinical Trial. Crit Rev Food Sci Nutr. 2019;59(20):3371–9. 10.1080/10408398.2018.1492900. 29993262

[149] Davison KM, Temple NJ. Cereal fiber, fruit fiber, and type 2 diabetes: Explaining the paradox. J Diabetes Complications. 2018;32(2):240–5. 10.1016/j.jdiacomp.2017.11.002. 29191432

[150] McRae MP. Dietary fiber intake and type 2 diabetes mellitus: An umbrella review of meta-analyses. J Chiropr Med. 2018;17(1):44–53. 10.1016/j.jcm.2017.11.002. PMC5883628 29628808

[151] Torres N, Avila-Nava A, Medina-Vera I, Tovar AR. Dietary fiber and diabetes. Science and Technology of Fibers in Food Systems. Cham: Springer; 2020. p. 201–18. 10.1007/978-3-030-38654-2_9.

[152] Seetaloo AD, Aumeeruddy MZ, Rengasamy Kannan RR, Mahomoodally MF. Potential of traditionally consumed medicinal herbs, spices, and food plants to inhibit key digestive enzymes geared towards diabetes mellitus management – A systematic review. South Afr J Bot. 2019;120:3–24. 10.1016/j.sajb.2018.05.015.

[153] Tanumihardjo SA, Valentine AR, Zhang Z, Whigham LD, Lai HJ, Atkinson RL. Strategies to increase vegetable or reduce energy and fat intake induce weight loss in adults. Exp Biol Med. 2009;234(5):542–52. 10.3181/0810-RM-293. 19234056

[154] Bassil MS, Gougeon R. Muscle protein anabolism in type 2 diabetes. Curr Opin Clin Nutr Metab Care. 2013;16(1):83–8. 10.1097/MCO.0b013e32835a88ee. 23196814

[155] Smith L, Fisher A, Hamer M. Television viewing time and risk of incident obesity and central obesity: the English longitudinal study of ageing. BMC Obes. 2015;2(1):12. 10.1186/s40608-015-0042-8. PMC4510888 26217527

[156] Kerley CP. A review of plant-based diets to prevent and treat heart failure. Card Fail Rev. 2018;4(1):1. 10.15420/cfr.2018:1:1. PMC5971679 29892479

[157] Sarker U, Oba S. Protein, dietary fiber, minerals, antioxidant pigments and phytochemicals, and antioxidant activity in selected red morph Amaranthus leafy vegetable. PLoS One. 2019;14(12):e0222517. 10.1371/journal.pone.0222517. PMC6907799 31830064

[158] Badimon L, Chagas P, Chiva-Blanch G. Diet and cardiovascular disease: Effects of foods and nutrients in classical and emerging cardiovascular risk factors. Curr Med Chem. 2019;26(19):3639–51. 10.2174/0929867324666170428103206. 28462707

[159] Tuso P. A plant-based diet, atherogenesis, and coronary artery disease prevention. Perm J. 2015;19(1):62–7. 10.7812/TPP/14-036. PMC4315380 25431999

[160] Satija A, Hu FB. Plant-based diets and cardiovascular health. Trends Cardiovasc Med. 2018;28(7):437–41. 10.1016/j.tcm.2018.02.004. PMC6089671 29496410

[161] Hu FB. Plant-based foods and prevention of cardiovascular disease: An overview. Am J Clin Nutr. 2003;78(3):544S–51S. 10.1093/ajcn/78.3.544S. 12936948

[162] Zamanian MY, Kujawska M, Nikbakhtzadeh M, Hassanshahi A, Ramezanpour S, Kamiab Z, et al. Carvacrol as a potential neuroprotective agent for neurological diseases: A systematic review article. CNS Neurol Disord – Drug Targets. 2021;20(10):942–53. 10.2174/1871527320666210506185042. 33970850

[163] Madigan M, Karhu E. The role of plant-based nutrition in cancer prevention. J Unexplored Med Data. 2018;3(11):9. 10.20517/2572-8180.2018.05.

[164] Niederreiter L, Adolph TE, Tilg H. Food, microbiome and colorectal cancer. Dig Liver Dis. 2018;50(7):647–52. 10.1016/j.dld.2018.03.030. 29705028

[165] Chang H, Lei L, Zhou Y, Ye F, Zhao G. Dietary flavonoids and the risk of colorectal cancer: An updated meta-analysis of epidemiological studies. Nutrients. 2018;10(7):950. 10.3390/nu10070950. PMC6073812 30041489

[166] Godos J, Bella F, Sciacca S, Galvano F, Grosso G. Vegetarianism and breast, colorectal and prostate cancer risk: An overview and meta-analysis of cohort studies. J Hum Nutr Diet. 2017;30(3):349–59. 10.1111/jhn.12426. 27709695

[167] Isabelle M, Lee BL, Lim MT, Koh W-P, Huang D, Ong CN. Antioxidant activity and profiles of common vegetables in Singapore. Food Chem. 2010;120(4):993–1003. 10.1016/j.foodchem.2009.11.038.

[168] Reuland EA, Al Naiemi N, Raadsen SA, Savelkoul PH, Kluytmans JA, Vandenbroucke-Grauls CM. Prevalence of ESBL-producing Enterobacteriaceae in raw vegetables. Eur J Clin Microbiol Infect Dis. 2014;33(10):1843–6. 10.1007/s10096-014-2142-7. PMC4182617 24848131

[169] Kowalczewski PŁ, Olejnik A, Świtek S, Bzducha-Wróbel A, Kubiak P, Kujawska M, et al. Bioactive compounds of potato (Solanum tuberosum L.) juice: From industry waste to food and medical applications. CRC Crit Rev Plant Sci. 2022;41(1):52–89. 10.1080/07352689.2022.2057749.

[170] Wang A, Islam MN, Johansen A, Haapalainen M, Latvala S, Edelenbos M. Pathogenic Fusarium oxysporum f. sp. cepae growing inside onion bulbs emits volatile organic compounds that correlate with the extent of infection. Postharvest Biol Technol. 2019;152:19–28. 10.1016/j.postharvbio.2019.02.010.

[171] Islam MN, Körner O, Pedersen JS, Sørensen JN, Edelenbos M. Analyzing quality and modelling mass loss of onions during drying and storage. Comput Electron Agric. 2019;164:104865. 10.1016/j.compag.2019.104865.

[172] Miedzianka J, Pęksa A, Nemś A, Drzymała K, Zambrowicz A, Kowalczewski P. Trypsin inhibitor, antioxidant and antimicrobial activities as well as chemical composition of potato sprouts originating from yellow- and colored-fleshed varieties. J Env Sci Heal Part B. 2020;55(1):42–51. 10.1080/03601234.2019.1657764. 31453739

[173] Kulczyński B, Gramza-Michałowska A. The profile of carotenoids and other bioactive molecules in various pumpkin fruits (Cucurbita maxima Duchesne) cultivars. Molecules. 2019;24(18):3212. 10.3390/molecules24183212. PMC6766813 31487816

[174] Kowalczewski PŁ, Zembrzuska J, Drożdżyńska A, Smarzyński K, Radzikowska D, Kieliszek M, et al. Influence of potato variety on polyphenol profile composition and glycoalcaloid contents of potato juice. Open Chem. 2021;19(1):1225–32. 10.1515/chem-2021-0109.

[175] Ersoy N, Bagci Y, Gok V. Antioxidant properties of 12 cornelian cherry fruit types (Cornus mas L.) selected from Turkey. Sci Res Essays. 2011;6:98–102.

[176] Lorts CM, Briggeman T, Sang T. Evolution of fruit types and seed dispersal: A phylogenetic and ecological snapshot. J Syst Evol. 2008;46:396–404. 10.3724/SP.J.1002.2008.08039.

[177] Thilakarathna SH, Rupasinghe HPV. Anti-atherosclerotic effects of fruit bioactive compounds: A review of current scientific evidence. Can J Plant Sci. 2012;92(3):407–19. 10.4141/cjps2011-090.

[178] Karasawa MMG, Mohan C. Fruits as prospective reserves of bioactive compounds: A review. Nat Products Bioprospect. 2018;8(5):335–46. 10.1007/s13659-018-0186-6. PMC6109443 30069678

[179] Coman V, Teleky B-E, Mitrea L, Martău GA, Szabo K, Călinoiu L-F, et al. Bioactive potential of fruit and vegetable wastes. Adv Food Nutr Res. 2020;91:157–225. 10.1016/bs.afnr.2019.07.001. 32035596

[180] Bodoira R, Maestri D. Phenolic compounds from nuts: Extraction, chemical profiles, and bioactivity. J Agric Food Chem. 2020;68(4):927–42. 10.1021/acs.jafc.9b07160. 31910006

[181] Rybicka I, Kiewlicz J, Kowalczewski PŁ, Gliszczyńska-Świgło A. Selected dried fruits as a source of nutrients. Eur Food Res Technol. 2021;247(10):2409–19. 10.1007/s00217-021-03802-1.

[182] Starowicz M, Piskuła M, Achrem–Achremowicz B, Zieliński H. Phenolic compounds from apples: reviewing their occurrence, absorption, bioavailability, processing, and antioxidant activity – a review. Pol. J. Food Nutr. Sci. 2020;70(4):321–36.

[183] Charalampopoulos D, Wang R, Pandiella S, Webb C. Application of cereals and cereal components in functional foods: A review. Int J Food Microbiol. 2002;79(1–2):131–41. 10.1016/S0168-1605(02)00187-3. 12382693

[184] Sidhu JS, Kabir Y, Huffman FG. Functional foods from cereal grains. Int J Food Prop. 2007;10(2):231–44. 10.1080/10942910601045289.

[185] Gani A, Wani SM, Masoodi FA, Hameed G. Whole-grain cereal bioactive compounds and their health benefits: A review. J Food Process Technol. 2012;3(3):146–56. 10.4172/2157-7110.1000146.

[186] Saleh ASM, Wang P, Wang N, Yang S, Xiao Z. Technologies for enhancement of bioactive components and potential health benefits of cereal and cereal-based foods: Research advances and application challenges. Crit Rev Food Sci Nutr. 2019;59(2):207–27. 10.1080/10408398.2017.1363711. 28846456

[187] Price RK, Welch RW. Cereal grains. Encyclopedia of Human Nutrition. Elsevier; 2013. p. 307–16. 10.1016/B978-0-12-375083-9.00047-7.

[188] Jiang C, Ci Z, Feng S, Wu S, Kojima M. Characteristics of functional components and antioxidant activity of 28 common beans. J Food Nutr Res. 2018;6(7):439–44. 10.12691/jfnr-6-7-3.

[189] Maphosa Y, Jideani VA. The role of legumes in human nutrition. Functional Food – Improve Health through Adequate Food. London, UK: InTech; 2017. 10.5772/intechopen.69127.

[190] Moreno-Valdespino CA, Luna-Vital D, Camacho-Ruiz RM, Mojica L. Bioactive proteins and phytochemicals from legumes: Mechanisms of action preventing obesity and type-2 diabetes. Food Res Int. 2020;130:108905. 10.1016/j.foodres.2019.108905. 32156360

[191] Muzquiz M, Varela A, Burbano C, Cuadrado C, Guillamón E, Pedrosa MM. Bioactive compounds in legumes: pronutritive and antinutritive actions. Implications for nutrition and health. Phytochem Rev. 2012;11(2–3):227–44. 10.1007/s11101-012-9233-9.

[192] Costantini M, Summo C, Centrone M, Rybicka I, D’Agostino M, Annicchiarico P, et al. Macro- and micro-nutrient composition and antioxidant activity of chickpea and pea accessions. Pol. J. Food Nutr. Sci. 2021;71(2):177–85.

[193] Chen D, Yang X, Yang J, Lai G, Yong T, Tang X, et al. Prebiotic effect of fructooligosaccharides from morinda officinalis on Alzheimer’s disease in rodent models by targeting the microbiota-gut-brain axis. Front Aging Neurosci. 2017;9:403. 10.3389/fnagi.2017.00403. PMC5727096 29276488

[194] Isic A. A study of flavonols in bok choy and their anti-cancer properties. PhD Thesis. Australia: Victoria University; 2017. https://vuir.vu.edu.au/37821.

[195] Kuriyama I, Musumi K, Yonezawa Y, Takemura M, Maeda N, Iijima H, et al. Inhibitory effects of glycolipids fraction from spinach on mammalian DNA polymerase activity and human cancer cell proliferation. J Nutr Biochem. 2005;16(10):594–601. 10.1016/j.jnutbio.2005.02.007. 16081275

[196] Deding U, Baatrup G, Christensen LP, Kobaek-Larsen M. Carrot intake and risk of colorectal cancer: A prospective cohort study of 57,053 danes. Nutrients. 2020;12(2):332. 10.3390/nu12020332. PMC7071341 32012660

[197] Tsai BC-K, Hsieh DJ-Y, Lin W-T, Tamilselvi S, Day CH, Ho T-J, et al. Functional potato bioactive peptide intensifies Nrf2-dependent antioxidant defense against renal damage in hypertensive rats. Food Res Int. 2020;129:108862. 10.1016/j.foodres.2019.108862. 32036911

[198] Cruz AB, Pitz H, da S, Veber B, Bini LA, Maraschin M, Zeni ALB. Assessment of bioactive metabolites and hypolipidemic effect of polyphenolic-rich red cabbage extract. Pharm Biol. 2016;54(12):3033–9. 10.1080/13880209.2016.1200633. 27436527

[199] Liu RH, Liu J, Chen B. Apples prevent mammary tumors in rats. J Agric Food Chem. 2005;53(6):2341–3. 10.1021/jf058010c. 15769178

[200] Mosa ZM, Khalil AF. The effect of banana peels supplemented diet on acute liver failure rats. Ann Agric Sci. 2015;60(2):373–9. 10.1016/j.aoas.2015.11.003.

[201] Kim H, Banerjee N, Barnes RC, Pfent CM, Talcott ST, Dashwood RH, et al. Mango polyphenolics reduce inflammation in intestinal colitis-involvement of the miR-126/PI3K/AKT/mTOR axis in vitro and in vivo. Mol Carcinog. 2017;56(1):197–207. 10.1002/mc.22484. PMC5053910 27061150

[202] Dkhil M, Al-Quraishy S, Abdel Moneim A. Effect of pomegranate (Punica granatum L.) juice and methanolic peel extract on testis of male rats. Pak J Zool. 2013;45:1343–9.

[203] Mandadi K, Ramirez M, Jayaprakasha GK, Faraji B, Lihono M, Deyhim F, et al. Citrus bioactive compounds improve bone quality and plasma antioxidant activity in orchidectomized rats. Phytomedicine. 2009;16(6–7):513–20. 10.1016/j.phymed.2008.09.001. 18930642

[204] Pérez-Beltrán YE, Becerra-Verdín EM, Sáyago-Ayerdi SG, Rocha-Guzmán NE, García-López EG, Castañeda-Martínez A, et al. Nutritional characteristics and bioactive compound content of guava purees and their effect on biochemical markers of hyperglycemic and hypercholesterolemic rats. J Funct Foods. 2017;35:447–57. 10.1016/j.jff.2017.06.022.

[205] Pipe EA, Gobert CP, Capes SE, Darlington GA, Lampe JW, Duncan AM. Soy protein reduces serum LDL cholesterol and the LDL cholesterol:HDL Cholesterol and apolipoprotein B:Apolipoprotein A-I ratios in adults with type 2 diabetes. J Nutr. 2009;139(9):1700–6. 10.3945/jn.109.109595. 19605528

[206] Sirato-Yasumoto S, Katsuta M, Okuyama Y, Takahashi Y, Ide T. Effect of sesame seeds rich in sesamin and sesamolin on fatty acid oxidation in rat liver. J Agric Food Chem. 2001;49(5):2647–51. 10.1021/jf001362t. 11368649

[207] Marineli R, da S, Lenquiste SA, Moraes ÉA, Maróstica MR. Antioxidant potential of dietary chia seed and oil (Salvia hispanica L.) in diet-induced obese rats. Food Res Int. 2015;76:666–74. 10.1016/j.foodres.2015.07.039. 28455051

[208] Ribeiro I, Costa C, Pereira V, Boaventura G, Chagas M. Effects of flaxseed flour on the lipid profile of rats submitted to prolonged androgen stimuli. Nutr Hosp organo la Soc Esp Nutr Parentery Enter. 2014;30:825–30. 10.3305/nh.2014.30.4.7301. 25335669

[209] Dotto JM, Chacha JS. The potential of pumpkin seeds as a functional food ingredient: A review. Sci Afr. 2020;10:e00575. 10.1016/j.sciaf.2020.e00575.

[210] Al-Attar AM. Therapeutic influences of almond oil on male rats exposed to a sublethal concentration of lead. Saudi J Biol Sci. 2020;27(2):581–7. 10.1016/j.sjbs.2019.12.035. PMC6997855 32210674

[211] Lu J-H, Hsia K, Lin C-H, Chen C-C, Yang H-Y, Lin M-H. Dietary supplementation with hazelnut oil reduces serum hyperlipidemia and ameliorates the progression of nonalcoholic fatty liver disease in hamsters fed a high-cholesterol diet. Nutrients. 2019;11(9):2224. 10.3390/nu11092224. PMC6770627 31540081

[212] Dias CCQ, Madruga MS, Pintado MME, Almeida GHO, Alves APV, Dantas FA, et al. Cashew nuts (Anacardium occidentale L.) decrease visceral fat, yet augment glucose in dyslipidemic rats. PLoS One. 2019;14(12):e0225736. 10.1371/journal.pone.0225736. PMC6907795 31830056

[213] Mohammed RR, Omer AK, Yener Z, Uyar A, Ahmed AK. Biomedical effects of Laurus nobilis L. leaf extract on vital organs in streptozotocin-induced diabetic rats: Experimental research. Ann Med Surg. 2021;61:188–97. 10.1016/j.amsu.2020.11.051. PMC7817776 33520200

[214] Bagheri S, Dashti-R MH, Morshedi A. Antinociceptive effect of Ferula assa-foetida oleo-gum-resin in mice. Res Pharm Sci. 2014;9:207–12. PMC4311286 25657791

[215] Alsuhaibani AMA. Effect of Nigella sativa against cisplatin induced nephrotoxicity in rats. Ital J Food Saf. 2018;7(2):7242. 10.4081/ijfs.2018.7242. PMC6036989 30046560

